# Cold homes are associated with poor biomarkers and less blood pressure check-up: English Longitudinal Study of Ageing, 2012–2013

**DOI:** 10.1007/s11356-016-6235-y

**Published:** 2016-02-13

**Authors:** Ivy Shiue

**Affiliations:** Faculty of Health and Life Sciences, Northumbria University, Newcastle upon Tyne, NE1 8ST, England UK; Alzheimer Scotland Dementia Research Centre, University of Edinburgh, Edinburgh, Scotland UK

**Keywords:** Adult health, Biomarker, Room temperature, Indoor environment, Cold home

## Abstract

It has been known that outdoor temperature influences seasonal fluctuation of blood pressure and cholesterol levels, but the role of indoor temperature has been less studied. Therefore, the aim of the present study was to examine the associations between indoor temperature and biomarkers in a countrywide and population-based setting. Data was retrieved from the English Longitudinal Study of Ageing, 2012–2013. Information on demographics, room temperature and a series of biomarkers measured in the blood and lung was obtained at household interviews. *t* test, chi-square test and a generalized linear model were performed cross-sectionally. Of 7997 older adults with the valid indoor temperature measurements, there were 1301 (16.3 %) people who resided in cold homes (<18 °C). Age was inversely associated with people who resided in cold homes or who tended not to have blood pressure check-up. Those who resided in cold homes had higher blood pressure readings, worse handgrip, lower vitamin D levels, higher cholesterol levels, higher insulin-like growth factor levels, higher haemoglobin levels, lower level of white blood cell count and worse lung conditions. One in six older adults aged 50 and above in England resided in cold homes and had poor biomarker values. For the future research direction, studies with a longitudinal approach to systematically monitor indoor temperature, biomarkers and health and wellbeing would be suggested. From the practice and policy perspectives, increasing health knowledge on the adverse effect of low indoor temperature on risks of cardiac and respiratory conditions, affording to the heating and re-designing of residential buildings to keep warm by using efficient energy, should be kept as priority.

## Introduction

### Evidence before this study

Cardiovascular disease has been persisting as the leading cause of illnesses and deaths globally (GBD 2013 Mortality and Causes of Death Collaborators 2015). With climate change, some regions could suffer from heat while some others might suffer from chill (Clearfield et al. 2014). There have also been observations on more hospital admissions on cold days than on warm days (Shiue et al. 2015, 2014a, b). Raised blood pressure (BP) and increased blood viscosity, as major risk contributors for cardiovascular disease, in moderate cold may be important causal factors in the increased winter morbidity and mortality due to heart attacks, strokes, respiratory symptoms, etc. (Collins 1986; Shiue and Shiue 2014). Apart from outdoor temperature, the indoor environment has gained attention for its effect on human health since we humans might spend much time indoors.

### Knowledge gap

It has been known that outdoor temperature influences the seasonal fluctuation of BP and cholesterol levels (Giaconi et al. 1989; Halonen et al. 2011). A recent pilot study of 26 older adults aged 70 and above has observed some change in physical performance in the circumstances of 20 and 30 °C (Stotz et al. 2014). However, the role of room temperature in biomarkers that could indicate many medical conditions has been much less studied.

### Study aim

Following this context, therefore, the aim of the present study was to investigate the prevalence of low indoor temperature (<18 °C) and to examine the associations between a cold home (low indoor temperature) and a series of biomarkers measured in the blood and lung in a countrywide and population-based setting with a focus on older adults since they could be more vulnerable than people in younger age groups.

## Methods

### Study sample

The English Longitudinal Study of Ageing (more details via http://www.elsa-project.ac.uk/) has been a countrywide, population-based, multi-year study since 1998. It is based on a representative sample of older adults aged 50+ living in private households in England, UK. A random sample was selected from the Health Survey for England (more details via http://www.hscic.gov.uk/healthsurveyengland) in different years. Data was collected continuously throughout the year (more details via http://www.esds.ac.uk/doc/5050/mrdoc/pdf/5050_Wave_3_Technical_Report.pdf#page=11). In the current analysis, the most recent study cohort, wave 6 in 2012–2013, with available data on demographics and objectively measured biomarkers and room temperature in older adults that was obtained by nurse interview was included.

### Variables and analyses

**Fig. 1:**
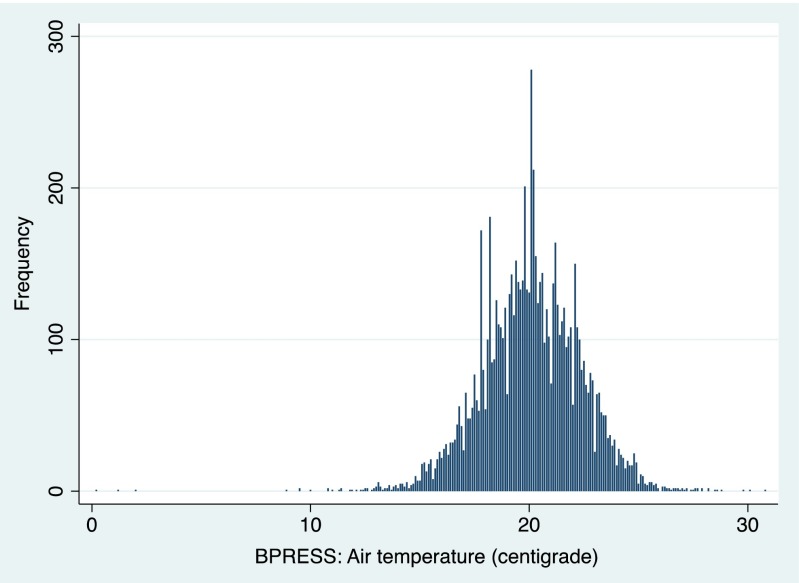
Distribution of room temperature in the studied households

Study exposure variable (*x* variable) was room temperature measured on the day of interview. It was measured in the room at the time BP was being taken. Figure 1 shows the distribution of room temperature from each household in the present study. Cold homes were defined as a room temperature below 18 °C, as recommended by the World Health Organization (more details via http://whqlibdoc.who.int/euro/pre-wholis/ICP_BSM_002%283%29.pdf). Study outcome variables (*y* variables) were a series of biomarkers that were measured in the blood and lung. Differences were examined by using *t* test or chi-square test while associations were examined by using a generalized linear model, with *P* < 0.05 considered statistically significant. Spike and scatter plots were also produced where needed. The statistical software STATA version 13.0 (STATA, College Station, Texas, USA; more details via http://www.stata.com/) was used to perform all the analyses.

## Results

Of 10,379 older adults aged 50 and above and included in the nurse interview data set, 7997 were with valid indoor temperature measurements. There were 1301 (16.3 %) people who resided in cold homes (<18 °C). Table 1 shows the differences and associations between cold homes and values of biomarkers measured in the blood and lung. Age was inversely associated with people who resided in cold homes or who tended not to have blood pressure check-up. Those who resided in cold homes had higher blood pressure readings, worse handgrip (of note: women tend to have lower grip strength than men have), lower vitamin D levels, higher cholesterol levels, higher insulin-like growth factor levels, higher haemoglobin levels, lower level of white blood cell count and worse lung conditions. The values in these biomarkers also showed linear associations with room temperature. However, there were no differences across sex, height and weight.

**Table 1:** Associations between cold homes and biomarkers

	≥18 °C (*n* = 6696, 83.7 %)	<18 °C (*n* = 1301, 16.3 %)	*P* value	Beta (95 %CI)
Sex			0.922	
Male	3001 (83.7 %)	585 (16.3 %)		
Female	3695 (83.8 %)	716 (16.2 %)		
Age			<0.001	
50–64	2440 (80.9 %)	575 (19.1 %)		
65–79	3249 (83.9 %)	622 (16.1 %)		
80+	1007 (90.6 %)	104 (9.4 %)		
Blood pressure check in the last year			<0.001	
Yes	5586 (84.6 %)	1016 (15.4 %)		
No	1096 (79.5 %)	283 (20.5 %)		
Dominant hand			0.104	
Right-handed	5887 (83.3 %)	1180 (16.7 %)		
Left-handed	654 (85.6 %)	110 (14.4 %)		
Height (cm)	165.3 ± 11.4	165.5 ± 14.0	0.504	–
Weight (kg)	77.5 ± 17.2	77.7 ± 17.8	0.607	–
Waist (cm)	95.3 ± 28.8	95.4 ± 31.5	0.904	–
Systolic (mmHg)	133.7 ± 18.7	136.8 ± 19.4	<0.001	−0.67 (−0.85 to −0.48)
Diastolic (mmHg)	74.2 ± 11.4	76.8 ± 11.0	<0.001	−0.61 (−0.72 to −0.50)
Pulse reading (bpm)	66.9 ± 19.6	67.3 ± 10.9	0.423	–
Mean arterial pressure (mmHg)	94.0 ± 12.2	96.8 ± 12.1	<0.001	−0.64 (−0.75 to −0.52)
Dominant hand grip (kg) (*n* = 7740)	27.9 ± 11.1	29.2 ± 11.3	<0.001	−0.31 (−0.42 to −0.21)
Non-dominant hand grip (kg) (*n* = 7740)	25.4 ± 10.5	26.5 ± 10.2	0.001	−0.29 (−0.39 to −0.18)
Total blood cholesterol level (mmol/l) (*n* = 6085)	5.5 ± 1.2	5.6 ± 1.1	0.011	−0.02 (−0.04 to −0.01)
Blood high-density lipoprotein level (mmol/l) (*n* = 6082)	1.7 ± 0.5	1.7 ± 0.5	0.328	–
Blood triglyceride level (mmol/l) (*n* = 6085)	1.5 ± 0.9	1.5 ± 0.8	0.082	–
Blood low-density lipoprotein level (mmol/l) (*n* = 6015)	3.2 ± 1.0	3.3 ± 1.0	0.002	−0.03 (−0.04 to −0.01)
Blood ferritin level (ng/ml) (*n* = 6085)	153.1 ± 144.8	151.0 ± 153.4	0.689	–
Blood C-reactive protein level (mg/l) (*n* = 6085)	3.5 ± 9.7	3.3 ± 6.1	0.500	–
Vitamin D level (unit) (*n* = 6071)	49.6 ± 23.7	44.1 ± 21.8	<0.001	0.98 (0.72-1-23)
Blood insulin-like growth factor level (nmol/l) (*n* = 6073)	16.3 ± 5.3	16.8 ± 5.5	0.007	−0.10 (−0.15 to −0.04)
Blood-glycated haemoglobin level (%) (*n* = 6011)	41.2 ± 8.6	40.9 ± 7.6	0.285	–
Blood glucose level (mmol/l)—fasting samples only (*n* = 3226)	5.4 ± 1.0	5.4 ± 0.8	0.857	–
Blood fibrinogen level (g/l) (*n* = 5967)	3.0 ± 0.5	3.0 ± 0.5	0.446	–
Blood haemoglobin level (g/dl) (*n* = 6019)	13.7 ± 1.3	13.9 ± 1.3	0.001	−0.05 (−0.07 to −0.04)
Blood mean corpuscular haemoglobin level (pg/cell) (*n* = 6019)	30.0 ± 1.8	30.1 ± 1.7	0.172	–
White blood cell count (× 10^9^ cells/l) (*n* = 6019)	6.5 ± 2.0	6.4 ± 1.9	0.005	0.08 (0.06–0.11)
Lung: forced vital capacity (*n* = 7009)	3.3 ± 1.1	3.4 ± 1.1	<0.001	−0.04 (−0.05 to −0.03)
Lung: forced expiratory flow (*n* = 7009)	2.4 ± 0.8	2.5 ± 0.8	0.0001	−0.03 (−0.04 to −0.02)
Lung: peak expiratory flow (*n* = 7009)	6.5 ± 2.4	6.7 ± 2.3	0.004	−0.06 (−0.09 to −0.04)

## Discussion

In the present study, poor biomarkers that were associated with low indoor temperature were blood pressure, mean atrial pressure, handgrip, blood low-density lipoprotein level, vitamin D level, blood insulin-like growth factor, blood haemoglobin level, white blood cell count and lung function values. Below is the existing literature, although very much limited, to compare with.

### Low temperature and health

Previously, it was observed that a low temperature could change cerebral blood volume and centre-of-foot pressure during walking in young adults (Demura et al. 2008), although the study sample was rather small (*n* = 18). While the handgrip of young and healthy college students (*n* = 12) was not significantly influenced by a low temperature at 18 °C (Barter and Freer 1984), older adults in the present study were affected; when room temperature dropped below 18 °C. Again, low temperature might not affect lung function in healthy young adults (*n* = 22), but it did affect older adults in the present study that is consistent with a previous study (Evans et al. 2005). For other biomarkers that were associated with the low indoor temperature among older adults in the present study, there was no other literature that could be compared and discussed.

On the other hand, temperature could profoundly influence growth of heterothermic vertebrates. This was previously observed in fish where temperature-induced variation in growth was associated with differences in systemic insulin-like growth factor I and local (i.e. muscle) insulin-like growth factor I mRNA levels (Luckenbach et al. 2007). Using human sample in the present study, the similar finding of blood insulin-like growth factor was also influenced by the low indoor temperature.

### Strengths and limitations

The present study has a few strengths. Firstly, it lies in its very large and representative study sample (countrywide and population-based) and in recent years. Secondly, it is also the first time to analyse the associations between room temperature and a series of biomarkers measured in the blood and lung in England, UK. However, there are also a few limitations that cannot be ignored. First, the room temperature was measured once in the room when biomarkers were being measured. Around the housing in other rooms, the temperature might be different. Second, the causality cannot be established due to the cross-sectional study design in nature. Therefore, the results could only reflect the situation at interview but not necessarily the whole time. Taken together, future studies keeping the strengths and overcoming the limitations mentioned above with a longitudinal approach to systematically monitor indoor temperature, biomarkers and health and wellbeing would be recommended.

### Future directions for research, practice and policy

One in six older adults aged 50 and above in England resided in cold homes, less had blood pressure check-up and were with poor biomarker values including blood pressure, mean atrial pressure, handgrip, blood low-density lipoprotein level, vitamin D level, blood insulin-like growth factor, blood haemoglobin level, white blood cell count and lung function. For the future research direction, studies with a longitudinal or experimental approach to systematically monitor indoor temperature, biomarkers and health and wellbeing in the whole population would be suggested. For clinical practice, maintaining indoor temperature at 18 °C or above should be implemented across all seasons throughout the year to optimize human health and wellbeing. In addition, intensive room heating that could decrease morning BP surge in winter and consequently hospital admissions due to cardiovascular and/or respiratory conditions would need to be designed via the heating technology (Saeki et al. 2013; Hashiguchi et al. 2004). Increasing health knowledge on the adverse effect of low indoor temperature on the higher risks of cardiac and respiratory conditions and assisting with job security in order to afford the heating and re-designing of residential buildings with more energy saving to keep the indoor efficiently warm should also be kept as a health priority. Since it was also observed that older adults in England who resided in cold homes below 18 °C had a lower level of vitamin D in the present study, encouraging people to spend more time in the sun while designing homes that could retain sunshine for longer time periods could be considered as well. These aspects would therefore need policymakers, clinicians, nursing staff, community workers, architects, urban planners and civil engineers to work together in order to protect the public’s health and to maintain wellbeing and quality of life when facing the extended life expectancy in the coming decades.
